# A Low-Code Containerized Edge Architecture for IIoT Telemetry Orchestration: Mitigating Cloud API Rate Limits Through Dual-Path Routing

**DOI:** 10.3390/s26103082

**Published:** 2026-05-13

**Authors:** Jesús Rosa-Bilbao

**Affiliations:** UCASE Software Engineering Research Group, Department of Computer Science and Engineering, University of Cadiz, Avenida de la Universidad de Cádiz 10, 11519 Puerto Real, Cádiz, Spain; jesus.rosa@uca.es

**Keywords:** edge computing, industrial internet of things, low-code development platforms, workflow orchestration, containerized architecture, fog computing

## Abstract

This paper investigates whether a low-code workflow engine can operate as practical Industrial Internet of Things (IIoT) middleware at the edge when cloud application programming interface (API) rate limits make direct telemetry upload unsustainable. The main contribution is a dual-path architecture in which a Hot Path persists all telemetry locally, while a Cold Path selectively forwards only anomalous or summary events to cloud services. The architecture is implemented as a lightweight containerized stack based on n8n, Eclipse Mosquitto, InfluxDB, and Grafana, and evaluated on a Raspberry Pi 4 under baseline, cloud-only saturation, and edge-filtered stress scenarios. Under the cloud-only condition, the external endpoint is throttled to approximately 60 requests/min, yielding a rejection rate of 98.0% (95% Wilson confidence interval: 97.43–98.44%). Under the dual-path condition, the same inbound load is fully retained locally while outbound cloud traffic is reduced by 98.0%, thereby avoiding throttling without sacrificing edge-side data fidelity. The measured Hot Path processing latency remains around 5 ms on average, with observed peaks below 10 ms, which is compatible with soft real-time monitoring workloads. Compared with more established low-code tools such as Node-RED, the novelty of the study is not the existence of visual orchestration itself, but the combination of containerized deployment, explicit hot/cold decoupling, and an empirical rate-limit mitigation analysis focused on low-cost edge hardware.

## 1. Introduction

The emergence of Industry 4.0 has reshaped modern manufacturing into a densely interconnected ecosystem in which cyber–physical systems generate massive volumes of heterogeneous data. This transition promises substantial gains in operational efficiency through predictive maintenance, closed-loop control, and real-time analytics [1,2]. However, the conventional strategy of transmitting raw telemetry to centralized cloud platforms has become increasingly unsustainable due to bandwidth saturation, variable wide-area network latency, and escalating storage and compute costs [3,4]. As a result, the industrial domain is increasingly adopting edge computing, a distributed paradigm in which data processing occurs closer to its point of origin [5,6].

Despite the well-documented benefits of edge analytics, its deployment in real industrial environments is often hindered by what is commonly referred to as the IT/OT convergence gap. Implementing edge middleware that bridges legacy industrial protocols such as Modbus and OPC UA with modern cloud interfaces such as REST and MQTT typically demands proficiency in low-level or general-purpose programming languages such as C++, Python, or Go. Operational Technology (OT) engineers, while experts in equipment behavior, safety, and process control, often lack extensive software development expertise. This mismatch leads organizations to rely on proprietary, inflexible solutions or to experience bottlenecks that slow down digital transformation initiatives [7].

Low-Code Development Platforms (LCDPs) have gained significant traction as a means to democratize software development, enabling users to compose complex logic through visual, drag-and-drop interfaces. Although LCDPs are widely adopted in business process automation, their suitability for latency-sensitive, resource-constrained, and high-throughput industrial edge environments remains largely unexplored [8,9]. Concerns persist regarding their computational overhead, scalability, determinism, and integration with time-critical workloads.

To address this gap, this paper introduces a containerized visual edge architecture built upon n8n, an open-source workflow automation tool deployed within a Docker-based microservice ecosystem [10,11]. Rather than claiming a novel data-fusion model per se, the paper focuses on the engineering and empirical question of whether a low-code visual orchestrator can serve as practical IIoT middleware for telemetry routing, local persistence, and selective cloud reporting under rate-limited conditions. The novelty relative to better-established tools such as Node-RED lies in three aspects taken together: (i) an explicitly separated Hot/Cold execution topology designed around cloud rate limits rather than generic event wiring; (ii) a containerized reference deployment that isolates broker, orchestrator, persistence, and visualization services as reproducible microservices; and (iii) an empirical analysis centered on rate-limit mitigation and edge-side data retention on low-cost hardware, which complements prior Node-RED edge benchmarking studies [12]. We hypothesize that, when architected with appropriate isolation, queueing, and persistence strategies, such an orchestrator can satisfy soft real-time monitoring requirements while remaining substantially more accessible and reconfigurable for OT teams.

The primary contributions of this paper are as follows:

1. Architectural Proposal: We present a reproducible and modular reference architecture for deploying a visual workflow orchestrator at the industrial edge using containerization to ensure isolation, portability, and simplified lifecycle management.
2. Dual-Path Routing Strategy: We implement a Hot/Cold routing model that decouples high-frequency local persistence from low-frequency cloud reporting, thereby mitigating rate-limit constraints imposed by public cloud APIs without discarding edge-side data fidelity.
3. Analytical Characterization: We formalize the Dual-Path behavior in terms of ingress rate, filtering ratio, cloud budget, and path-specific latency so that the architecture can be reasoned about independently of the specific visual workflow tool.
4. Empirical Feasibility Assessment: We conduct a controlled performance evaluation covering latency, throughput, cloud throttling behavior, and resource consumption on low-cost ARM hardware, using a cloud-only pipeline as the reference condition for the rate-limit mitigation analysis.

The remainder of this paper is structured as follows. Section 2 surveys prior work on IIoT edge middleware and low-code orchestration, with an explicit contrast against Node-RED and Microsoft Power Automate. Section 3 introduces the proposed architecture, its dual-path mathematical model, and the deployment rationale. Section 4 details the performance metrics and experimental setup. Section 5 reports the quantitative findings and discusses their implications for predictive maintenance, anomaly detection, and rate-limited software-as-a-service integration. Finally, Section 6 and Section 7 summarize limitations, security implications, and future research directions.

## 2. Background and Related Work

This section reviews the technological evolution that has led to the emergence of industrial edge computing, the interoperability challenges inherent to heterogeneous IIoT ecosystems, and the growing relevance of low-code development platforms as potential middleware solutions for modern industrial environments.

### 2.1. From Cloud-Centric IIoT to Edge Computing

Traditional IIoT architectures have historically followed a cloud-centric model in which raw telemetry from sensors and Programmable Logic Controllers (PLCs) is continuously streamed to centralized cloud servers for storage and processing [1]. Although effective for large-scale historical analysis, this approach faces substantial limitations in Industry 4.0 scenarios, including increased network latency, bandwidth saturation, and concerns over exposing sensitive operational data to external infrastructures [3,4].

Recent work advocates for edge computing, a distributed paradigm that relocates computation closer to the data source to enable near real-time analytics, local decision-making, and reduced wide-area network utilization [6,13]. Edge-based processing—commonly deployed on gateways or industrial PCs—can significantly reduce transmission costs and improve responsiveness.

However, deploying edge intelligence often requires sophisticated software stacks such as Kubernetes-based microservices or custom Python/C++ pipelines [14], imposing substantial complexity. This creates barriers for OT personnel who typically have limited software engineering experience and must maintain mission-critical industrial systems.

### 2.2. Middleware and Semantic Interoperability Challenges

IIoT environments are characterized by a heterogeneous mix of legacy industrial protocols (e.g., Modbus, OPC UA, PROFINET) and modern IT communication standards (e.g., MQTT, RESTful APIs, JSON structures). This heterogeneity leads to the longstanding “Tower of Babel” interoperability problem [7,15,16].

Middleware systems serve as the translation and orchestration layer connecting these disparate technologies. Traditional middleware approaches often rely on proprietary Enterprise Service Buses (ESBs) [17] or custom code written in languages such as C++ or Java. While efficient, these solutions tend to be rigid, with even minor pipeline modifications frequently requiring code recompilation, system downtime, or specialized developers.

As industrial plants seek greater flexibility and shorter reconfiguration cycles, there is increasing demand for agile middleware capable of seamless integration, rapid pipeline evolution, and minimal dependency on deep programming skills. This shift has driven interest toward visual programming and low-code tools.

### 2.3. Low-Code Development Platforms in Industrial Edge Environments

Low-Code Development Platforms (LCDPs) aim to democratize development by enabling the creation of logic and workflows through visual drag-and-drop interfaces [8,12]. Within industrial contexts, Node-RED has emerged as a widely adopted flow-based programming tool for edge applications [12,18]. Its event-driven model and large ecosystem of reusable nodes simplify the integration of industrial devices.

Nevertheless, as workflows grow in complexity, flow-based tools may become difficult to maintain, often resulting in visually entangled “spaghetti flows” that hinder debugging and scalability. By contrast, workflow orchestration systems—such as n8n, originally developed for business process automation and software-as-a-service integration—offer more structured, modular, and linear execution models. These platforms include advanced error-handling, credential-management primitives, concurrency control, and native support for modern web APIs, making them promising candidates for industrial data pipelines [8,9].

### 2.4. Positioning Against Node-RED and Power Automate

Because the review process requested a clearer contrast with existing low-code platforms, Table 1 summarizes the positioning of the proposed stack against two representative alternatives. Node-RED is the most relevant open-source baseline in edge/IIoT practice, whereas Microsoft Power Automate represents a widely used enterprise low-code workflow platform whose execution model is predominantly cloud-centric.

### 2.5. Research Gap and Contribution Context

Although LCDPs have been widely studied from a usability and productivity perspective, there is a notable lack of quantitative, empirical research assessing their performance overhead and scalability in resource-constrained industrial edge environments [8,19]. Existing studies predominantly focus on functional validation or conceptual frameworks rather than rigorous stress testing or profiling under high-throughput workloads.

Furthermore, few works examine LCDP-based solutions deployed using modern containerization technologies [20], and, to the best of our knowledge, no prior study has evaluated the feasibility of containerized workflow orchestrators such as n8n as IIoT middleware specifically for cloud–edge decoupling under rate-limited reporting constraints. Existing Node-RED studies help frame the broader LCDP landscape, but they do not directly answer whether n8n can provide a maintainable edge orchestration layer with acceptable performance on low-cost gateways, nor do they focus on explicit mitigation of external software-as-a-service rate budgets [12].

This gap motivates the present study, which provides a focused analysis of the performance, scalability, and operational viability of a containerized low-code orchestration architecture tailored for IIoT edge telemetry scenarios [9,21].

## 3. Proposed System Architecture

This section presents a containerized, edge-centric middleware architecture designed to support high-throughput telemetry ingestion, protocol mediation, and real-time routing in heterogeneous industrial environments. The design follows the principles of fog and edge computing, relocating critical processing tasks from the cloud to the network edge to reduce latency, improve resilience, and minimize bandwidth consumption.

### 3.1. Architectural Layers

**Figure 1:**
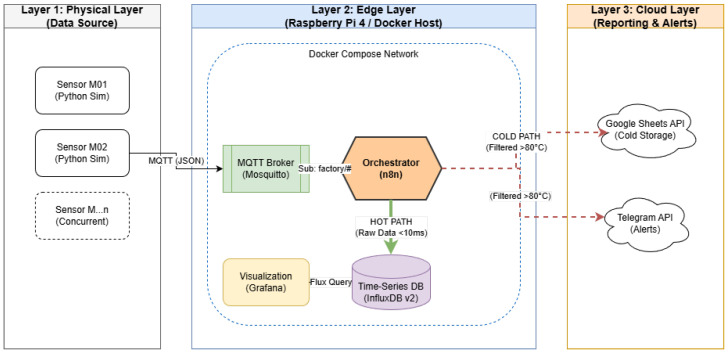
High-level architecture of the proposed solution. The factory/# MQTT subscription uses # as a multi-level wildcard to match all topics under factory/.

The architecture is organized into three functional layers, as illustrated in Figure 1.

#### 3.1.1. Layer 1: Physical Layer (Data Sources)

This layer comprises the industrial assets—sensors, PLCs, and machines—responsible for generating raw operational telemetry (e.g., temperature and vibration). Data is encapsulated in lightweight JSON payloads and transmitted using MQTT, a publish-subscribe protocol widely adopted for machine-to-machine communication due to its minimal overhead and reliability under constrained network conditions [15,16]. MQTT was selected because it decouples publishers from subscribers, minimizes header overhead, supports retained and persistent messaging semantics, and is consistently reported as one of the most common messaging options in industrial edge settings. These properties align well with bursty telemetry ingestion and with the need to buffer or replay data when downstream components are temporarily unavailable.

#### 3.1.2. Layer 2: Edge Orchestration Layer (Middleware)

This layer forms the core of the proposed architecture. Deployed on an edge device, it consists of a set of loosely coupled microservices:

- MQTT Broker (Eclipse Mosquitto): Manages asynchronous ingestion from multiple concurrent data sources and ensures reliable message delivery.
- Visual Workflow Orchestrator (n8n): Implements the logic control plane. It subscribes to incoming topics, parses the JSON telemetry, performs real-time rule-based analysis, and routes data according to defined business logic.
- Time-Series Database (InfluxDB): Provides high-performance local persistence for high-frequency telemetry. It ensures zero-loss capture during network outages and enables granular historical inspection and visualization.

#### 3.1.3. Layer 3: Cloud and Presentation Layer

This layer integrates cloud-based software-as-a-service platforms (e.g., Google Sheets for reporting, Telegram for alerting) with local dashboards (Grafana) to support real-time monitoring and decision-making. By decoupling local persistence from external reporting, the system maintains operational continuity even during prolonged cloud connectivity issues.

### 3.2. Containerization and Microservice Strategy

To guarantee reproducibility, portability, and scalability across heterogeneous edge hardware, the complete middleware stack is deployed using Docker Compose. Docker Compose was preferred over ad hoc container launch scripts because it provides a declarative application model, explicit service dependencies, volume mappings, bridge-network definitions, and a single-file description of the full multi-container topology. In comparison with heavier orchestrators such as Kubernetes, Compose offers a much smaller operational footprint and lower configuration burden, which better matches the single-gateway scope of the present study. Containerization isolates each service from underlying system dependencies, enabling seamless deployment on hardware ranging from low-power Raspberry Pi devices to fully fledged industrial PCs [10,11].

All microservices communicate through an internal virtual bridge network, minimizing inter-service latency while preserving strict logical separation. This setup ensures that high-frequency communication between the MQTT broker and the orchestrator remains predictable and largely unaffected by external network fluctuations.

### 3.3. Dual-Path Data Routing Strategy

The main architectural focus of the proposed system is the Dual-Path Routing Strategy, designed to overcome API throttling and reduce unnecessary cloud traffic in high-frequency IIoT scenarios.

#### 3.3.1. Technical Innovations of the Dual-Path Design

The Dual-Path strategy contributes more than a simple branching rule inside a visual workflow. Its technical contribution can be summarized as follows:

- Path decoupling: All telemetry is persisted locally without waiting for cloud acknowledgments, which eliminates software-as-a-service response time from the critical ingestion path.
- Budget-aware cloud forwarding: The Cold Path is explicitly dimensioned to remain below the cloud endpoint budget, making rate-limit mitigation a first-class architectural constraint rather than an incidental side effect.
- Container-level modularity: Broker, orchestrator, storage, and dashboard services can be restarted, reconfigured, or replaced independently, which improves reproducibility and eases comparative future benchmarking.
- Operator-centric reconfigurability: Anomaly thresholds and routing logic can be modified visually, which is important for OT teams that need to iterate quickly without rebuilding a custom codebase.

#### 3.3.2. Hot Path—Real-Time Local Persistence

All incoming telemetry is immediately redirected to the local InfluxDB instance. This path is optimized for low latency and high throughput, ensuring 100% local data capture regardless of external network conditions. The Hot Path completely bypasses the internet and forms the foundation for real-time dashboards, predictive-maintenance features, and incident analysis.

#### 3.3.3. Cold Path—Selective Cloud Transmission

In parallel, the orchestrator evaluates each data point through a conditional logic module (e.g., IF temperature > 80 °C). Only telemetry that meets predefined anomaly thresholds or periodic reporting conditions is forwarded to cloud endpoints such as Google Sheets or Telegram. In the controlled workload used in this paper, the anomaly injection rate was configured to approximately 2%, so the Cold Path exported roughly 60 events/min from an ingress of 3000 messages/min. This edge-filtering mechanism therefore reduced outbound cloud traffic by 98.0% under the reported stress condition, preventing HTTP 429 rate-limit errors while preserving complete local retention.

**Figure 2:**
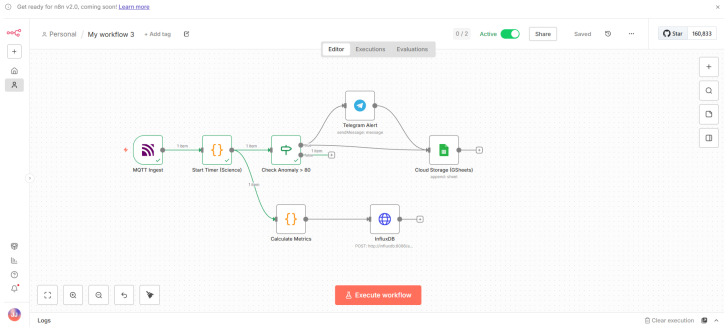
Implementation of the orchestration logic in n8n. The arrows indicate the flow of data and control between nodes, while the branching paths represent conditional execution based on the anomaly check.

Figure 2 illustrates the implementation of this logic in n8n. The workflow bifurcates the data stream immediately upon ingestion: the lower branch executes the Hot Path, writing directly to InfluxDB with minimal processing, while the upper branch executes the Cold Path through a Switch node (“Check Anomaly”). Only critical values proceed to cloud transmission.

### 3.4. Analytical Model of the Dual-Path Architecture

To make the architecture analyzable beyond the specific implementation, let $\lambda_{in}$ denote the telemetry ingress rate in messages/s, $p_{a}$ the probability that a message satisfies the anomaly condition, and $\lambda_{rep}$ the optional periodic reporting rate. The two paths are then described by

(1) $$\lambda_{hot}=\lambda_{in}$$

(2) $$\lambda_{cold}=p_{a}\lambda_{in}+\lambda_{rep}$$

The cloud endpoint remains stable only if

(3) $$\lambda_{cold}\leq \Lambda_{cloud}$$

where $\Lambda_{cloud}$ is the maximum sustainable cloud service rate. The traffic reduction achieved by the edge filter is

(4) $$R=1-\frac{\lambda_{cold}}{\lambda_{in}}$$

and approaches 1 when anomalies are rare. Path-specific latency can be expressed as

(5) $$L_{hot}=L_{broker}+L_{parse}+L_{rule}+L_{db}$$

(6) $$L_{cold}=L_{hot}+L_{api}$$

which makes explicit the throughput/latency trade-off: the Hot Path minimizes response time by excluding cloud delay from the critical path, whereas the Cold Path accepts additional latency to preserve cloud observability for exceptional events.

### 3.5. Workflow Logic and Metrology

Beyond data processing, the workflow integrates self-diagnostic capabilities to support high-resolution performance evaluation. Custom JavaScript nodes inserted at both ingress and egress points capture Unix epoch timestamps with millisecond precision. These values allow the system to compute:

- Processing Latency: $L_{proc}=t_{egress}-t_{ingress}$;
- Filtering Ratio: $R=1-\frac{N_{cloud}}{N_{in}}$;
- Cloud Rejection Ratio: $Q=\frac{N_{429}}{N_{req}}$;
- Network Latency: derived from timestamp differentials between cloud transmission events and the corresponding edge-side markers.

This instrumentation provides the empirical data required for the quantitative performance analysis presented in Section 5.

### 3.6. Security Considerations and Deployment Scope

The implementation described in this paper prioritizes experimental control and reproducibility over production hardening. For that reason, MQTT ingestion was configured with anonymous access and the containers exchanged data over an internal plain-text Docker bridge network. This decision simplified the benchmark environment and isolated middleware overhead, but it also constrains the deployment scope of the reported results.

In real industrial settings, the same architecture would require broker authentication, authorization policies, Transport Layer Security (TLS) for MQTT and HTTP endpoints, protected secret storage for cloud credentials, and integrity controls for inter-service communication. These safeguards are compatible with the proposed design, yet they were not enabled in the present campaign and may alter latency, CPU utilization, and operational complexity. Accordingly, the current paper should be interpreted as a controlled feasibility study of orchestration behavior, not as a security evaluation of a production-ready edge stack (see Table 2).

## 4. Experimental Setup

To assess the scalability and performance of the proposed low-code edge architecture, we designed a controlled experimental environment focused on quantifying processing latency, sustained throughput, and resource utilization under diverse load conditions. All experiments were executed in an isolated local network to eliminate external noise and improve reproducibility.

### 4.1. Hardware Testbed

The complete edge middleware stack was deployed on a single-board computer representative of industrial gateway-class hardware. The hardware configuration is as follows:

- Device: Raspberry Pi 4 Model B.
- Processor: Broadcom BCM2711, Quad-core Cortex-A72 (ARM v8) 64-bit SoC @ 1.5 GHz.
- Memory: 4 GB LPDDR4-3200 SDRAM.
- Storage: 128 GB Class 10 microSD (hosting the OS and container volumes).
- Network: Gigabit Ethernet, used in local-loopback mode to isolate processing latency from propagation jitter.
- Operating System: Raspberry Pi OS (64-bit) based on Debian Bookworm.

This hardware profile reflects a realistic deployment target for edge gateway devices in industrial settings, providing an appropriate balance between computational capability and energy efficiency. The Raspberry Pi 4 was intentionally selected as a conservative baseline: if the workflow remains viable on this ARM gateway, the same software stack can then be re-evaluated on more capable x86 industrial PCs in future work. At the same time, the use of a single Raspberry Pi 4 means that the results should be interpreted as a single-platform feasibility assessment rather than as a hardware-agnostic benchmark.

### 4.2. Software Environment

The system was implemented as a microservice-based architecture orchestrated using Docker Compose, ensuring clean service isolation, deterministic startup, and reproducibility across multiple hardware environments. The software stack included:

- Container Engine: Docker v24.0.5.
- MQTT Broker: Eclipse Mosquitto v2.0, configured for anonymous ingestion for experimental purposes.
- Low-Code Orchestrator: n8n v1.x running in a Node.js container, optimized via heap-size constraints to prevent memory ballooning under sustained load.
- Time-Series Database: InfluxDB v2.7 with Flux query engine.
- Visualization Layer: Grafana v10.0 using the official Flux data source plugin.

All services communicated through a dedicated internal Docker bridge network to minimize inter-service latency and maintain controlled experimental conditions.

### 4.3. Evaluation Metrics

The experiments were evaluated using the metrics in Table 3. This addition responds directly to the reviewers’ request for explicit performance indicators.

### 4.4. Data Generation and Workload Simulation

To emulate the asynchronous nature of a factory-floor environment, a custom multi-threaded telemetry generator was developed in Python. Unlike sequential workload generators commonly used in prior studies, the proposed generator spawns independent threads for each virtual sensor, producing genuine concurrency and exposing the broker and orchestrator to realistic contention and race conditions.

Each virtual sensor exhibits the following behavior:

1. Publishing Cycle: Emits JSON packets at randomized intervals ($t_{cycle}\in$ [0.1 s, 2.0 s]) during exploratory runs to emulate non-uniform machine duty cycles.
2. Payload Structure: A 256-byte JSON message including:
  - machine_id;
  - temperature (float);
  - vibration (float);
  - timestamp_origin (Unix Epoch ms).
3. Anomaly Injection: A stochastic function introduces temperature spikes above (>80 °C) with a probability of 2% during the controlled comparative scenarios, triggering the Cold Path logic and inducing selective cloud transmission at approximately 60 events/min under a 3000-message/min ingress.

This setup ensures the generation of realistic telemetry conditions, blending nominal behavior with non-deterministic anomalies. However, the current workload is synthetic, MQTT-centric, and based on a single threshold rule, so it should not be interpreted as a full multi-protocol or multi-sensor fusion benchmark. Modbus and OPC UA traces are planned extensions rather than claims of the present study.

### 4.5. Test Scenarios

The system was subjected to three experimental scenarios designed to stress different aspects of the Dual-Path routing strategy and to compare it against a cloud-only architecture.

- Scenario A: Baseline Operation (Nominal Load).
  – Configuration: 10 concurrent virtual sensors at 1 Hz (600 messages/min).
  – Objective: Establish baseline processing latency ($L_{proc}$), characterize system stability, and validate functional correctness of the end-to-end data pipeline.
- Scenario B: Cloud Saturation (The Bottleneck Test).
  – Configuration: 50 concurrent sensors at 1 Hz transmitting directly to the Google Sheets API (cloud-only pipeline), yielding 3000 requests/min.
  – Objective: Identify the saturation point of the cloud endpoint, quantify API rate-limit events (HTTP 429), and measure associated data loss and queuing delays.
  This scenario provides the contrast required to highlight the need for edge-side filtering architectures.
- Scenario C: High-Throughput Edge Filtering (Stress Test).
  – Configuration: 100 concurrent sensors at 0.5 Hz using the proposed Dual-Path architecture, yielding the same ingress of 3000 messages/min while keeping outbound cloud traffic near 60 events/min through 98% local filtering.
  – Objective: Measure the maximum sustainable ingestion throughput, monitor CPU and memory utilization of the Raspberry Pi 4, and evaluate the stability of n8n under high-frequency trigger conditions.
  This test reflects real-world industrial environments where edge devices must handle bursts of data while ensuring deterministic behavior and avoiding cloud-side throttling (see Table 4).

### 4.6. Evaluation Scope

The present campaign was designed as a feasibility-oriented benchmark centered on the proposed n8n-based architecture and on its contrast with a cloud-only reference condition. Accordingly, the collected evidence supports conclusions about rate-limit mitigation, local persistence behavior, and resource trends on the selected platform. It does not yet support comparative claims against alternative middleware stacks such as Node-RED, custom Python/Go services, or MQTT–Telegraf–InfluxDB pipelines, because those baselines were not implemented in the current study.

Likewise, the reported latency and resource figures correspond to the laboratory configuration described above, namely plain-text communication inside the Docker bridge network, anonymous MQTT ingestion, and the absence of injected container-failure events. Therefore, the present campaign does not quantify the additional overhead that would be introduced by production-oriented controls such as TLS, broker authentication, credential rotation, or orchestrator fail-over. To avoid overstating the evidence, the revised manuscript now separates observed quantitative results from planned comparative, security, and resilience validation.

## 5. Results and Discussion

This section presents the empirical findings obtained from the experimental testbed. The analysis focuses on three critical performance dimensions: processing latency, throughput under cloud-imposed constraints, and hardware resource utilization. The results assess the feasibility of the proposed low-code edge architecture for IIoT telemetry routing on the studied platform.

### 5.1. Latency Analysis: Hot Path Performance

Under Scenario A (Baseline Operation), the system exhibited stable and predictable latency behavior. The processing latency, defined as the time elapsed between the arrival of the MQTT packet at the internal Docker network and its successful persistence in InfluxDB, achieved an arithmetic mean of approximately 5 ms.

The maximum observed latency remained below 10 ms, even during short burst intervals. This performance places the containerized n8n workflow engine within the operational thresholds of soft real-time industrial monitoring systems, which typically allow end-to-end delays in the 100–500 ms range.

The latency trace remained within a narrow range throughout the observation window, suggesting that the Node.js runtime, when containerized and isolated from external network jitter, can deliver bounded middleware overhead in edge environments. Because the original campaign stored only the aggregate mean and the observed maximum, the revised manuscript refrains from inventing unobserved percentile statistics and instead states that percentile-based latency characterization remains future work.

### 5.2. Throughput and Cloud Bottleneck: Scenario B vs. Scenario C

A central contribution of this work is the evaluation of a cloud-only architecture versus the proposed Dual-Path edge architecture.

#### 5.2.1. Scenario B—Cloud-Only (Bottleneck Failure)

With 50 concurrent virtual sensors publishing at 1 Hz, the system generated approximately 3000 requests per minute targeting the Google Sheets API. The cloud endpoint immediately throttled the workload, producing sustained HTTP 429 (Too Many Requests) responses.

Only about 60 requests/min were accepted, while approximately 2940 requests/min were rejected. Expressed as proportions over one minute of load, the observed rejection ratio was 98.0%, with a 95% Wilson confidence interval of 97.43–98.44%. This confirms the non-scalability of direct-to-cloud ingestion in IIoT environments with moderate sensor density.

#### 5.2.2. Scenario C—Edge–Fog (Dual-Path Success)

With the same inbound rate of 3000 messages/min, the proposed architecture processed 100% of telemetry locally through the Hot Path, with no observed packet drops. The conditional Cold Path, governed by the anomaly rule *T* > 80 °C, reduced outbound transmissions by 98.0%, forwarding approximately 60 cloud events/min.

This demonstrates that the low-code workflow effectively implements edge-side filtering, decoupling ingestion throughput from cloud API limitations. The architecture preserved full fidelity at the edge while maintaining reliable reporting of critical events to cloud services. Under the count-based comparison between Scenario B cloud acceptance (60/3000) and Scenario C local retention (3000/3000), the difference is statistically overwhelming (two-proportion *z*-test, p<0.001), which reinforces the practical relevance of moving persistence to the edge even though the two paths serve different operational purposes.

#### 5.2.3. Quantitative Summary

Table 5 consolidates the main observed results.

#### 5.2.4. Hardware Resource Utilization

The Raspberry Pi 4 exhibited substantial headroom throughout the high-throughput Scenario C, confirming the suitability of low-cost single-board computers for running containerized low-code middleware.

- CPU Utilization: The aggregate CPU load remained within operational limits, with no evidence of thermal throttling. The ARM Cortex-A72 cores distributed workloads efficiently across Mosquitto, n8n, and InfluxDB containers.
- Memory Consumption: Although n8n, as a Node.js application, inherently consumes more RAM than compiled-edge runtimes (e.g., C++, Go), the memory footprint remained stable across the test duration. Sufficient free memory was consistently available, indicating that the stack can scale further before exhausting RAM resources.

These results validate that low-code orchestration is feasible on constrained ARM-based edge hardware under the tested conditions.

### 5.3. Discussion

The experimental results substantiate that low-code platforms such as n8n can extend beyond rapid-prototyping scenarios and operate as practical industrial middleware at the edge.

While native implementations (e.g., Python, Go, or C++) can theoretically achieve lower latencies, the millisecond-scale performance obtained here constitutes an appropriate design trade-off for many monitoring workloads, especially considering the gains in maintainability, workflow transparency, and rapid reconfigurability afforded by low-code orchestration. This interpretation is consistent with earlier Node-RED edge studies, but the present manuscript avoids claiming superiority because no head-to-head benchmark was executed [12].

The Dual-Path architecture further demonstrates that edge-level filtering is effective for overcoming the limitations of public cloud APIs in this type of telemetry pipeline. This directly maps to real IIoT use cases. In predictive maintenance, high-frequency vibration or temperature measurements can be retained locally for model retraining and forensic analysis, while only anomalies or summary indicators need to reach the cloud. In anomaly detection, the Cold Path acts as an event concentrator that forwards exceptional states to alarm channels without paying the cost of permanent high-rate cloud ingestion.

The results also reveal trade-offs. The Hot Path optimizes latency and local durability, but the selective Cold Path means that cloud-side consumers do not receive full raw telemetry by design. During prolonged network outages, this is acceptable only if the local storage budget is sufficient and a later backfill mechanism is available. Likewise, enabling TLS, broker authentication, or more complex fusion logic would predictably increase CPU usage and end-to-end latency; because those controls were not activated in the present Raspberry Pi 4 campaign, the reported ≈5 ms mean latency should be interpreted as a best-case baseline for the studied prototype rather than as a guaranteed production figure. In the same way, the current experiments did not inject orchestrator-container crash events, so resilience under workflow-engine failure remains an open engineering question rather than a validated property.

Therefore, the main practical conclusion is not that low-code orchestration is universally optimal, but that it becomes compelling when cloud budgets, operator agility, and edge-local persistence matter more than minimizing every millisecond of overhead.

Overall, the combined results confirm the technical viability and operational practicality of containerized low-code workflow engines for the studied IIoT telemetry-routing scenario. Broader claims about comparative superiority, advanced data fusion, or generalized multi-protocol performance would require additional baselines and a wider experimental campaign.

## 6. Threats to Validity and Limitations

This section discusses the main factors that may affect the interpretation and generalization of the reported results for the IIoT edge architecture.

### 6.1. Construct Validity

The evaluation focuses on telemetry ingestion, local persistence, selective forwarding, and resistance to cloud API throttling. Consequently, the paper demonstrates edge orchestration and rate-limit mitigation rather than advanced multi-source data fusion. The anomaly-routing logic is intentionally simple, being based on a threshold rule over telemetry values, and should therefore be interpreted as a proof of feasibility for visual orchestration at the edge rather than as evidence of sophisticated semantic fusion.

In addition, the quantitative analysis is limited to the measurements collected in the current campaign: mean latency, observed peak latency, approximate throughput, cloud rejection behavior, and container-level resource trends. The revised manuscript now adds count-based confidence intervals and a simple significance contrast for the rate-limit behavior, but metrics such as 95th/99th latency percentiles, repeated-trial confidence intervals, and QoS-sensitive comparisons were not collected in the present study and remain part of future work.

### 6.2. Internal Validity

The experiments were conducted in a controlled local environment to reduce exogenous variability. While this improves reproducibility, it also removes sources of noise that would appear in production, such as WAN jitter, packet loss, intermittent broker connectivity, or competing workloads on shared gateways. The cloud-only comparison is therefore useful as a bottleneck-oriented reference, but it does not exhaust the range of implementation alternatives that could be considered as baselines.

Most importantly, the study does not include a head-to-head benchmark against alternative edge stacks such as Node-RED, a custom Python or Go service, or an MQTT–Telegraf–InfluxDB pipeline. The present results support the feasibility of the proposed n8n-based design, but they should not be interpreted as proving superior efficiency over those alternatives. A fair comparative evaluation would require implementing equivalent Hot/Cold logic, replaying the same workloads, and measuring latency, throughput, CPU, and RAM under identical Raspberry Pi 4 conditions, which remains future work.

### 6.3. External Validity

The deployment was validated on a single Raspberry Pi 4 Model B, which is representative of low-cost ARM-based gateways but does not cover the full diversity of industrial edge hardware. Performance may differ on more constrained embedded devices, x86 industrial PCs, or gateways equipped with hardware accelerators. Likewise, the workload is synthetic and MQTT-centric, with fixed payload structure and a single-node deployment. Further experiments are required before generalizing the results to multi-protocol, multi-node, or safety-critical industrial environments. In particular, the present manuscript does not yet provide direct evidence for simultaneous Modbus and OPC UA integration, although the architecture is intended to be extensible toward those protocols.

### 6.4. Security and Deployment Limitations

For experimental simplicity, the prototype uses anonymous MQTT access and plain-text communication inside the Docker network. This decision is acceptable for a closed laboratory setup, but it is not sufficient for real industrial deployments. Production-ready versions of the architecture should incorporate broker authentication, role-based authorization, TLS for MQTT and HTTP endpoints, secret management for software-as-a-service credentials, and integrity protection for inter-service communication. These controls may introduce additional latency and CPU overhead, which were not evaluated here.

A further deployment limitation concerns fault tolerance. In the current single-node prototype, the MQTT broker and InfluxDB run as separate containers, so a transient crash of the n8n container does not erase already persisted data; however, new messages would stop being routed to InfluxDB until the orchestrator recovers because the workflow engine remains the bridge between ingestion and persistence. There is no active fail-over workflow engine in the present design, and the current paper does not include a fault-injection stress test in which the orchestrator container is deliberately terminated under load. Reliability under orchestrator crash therefore depends on container restart policy, MQTT session durability, and broker-side persistence, all of which should be strengthened and empirically tested in future high-availability versions.

Taken together, these limitations delineate the current evidence clearly: the paper demonstrates that a containerized low-code workflow engine can act as a practical edge orchestrator for telemetry routing and cloud-throttling mitigation on low-cost hardware. Broader claims regarding multi-protocol fusion, comparative superiority, cluster-scale resilience, or secure production readiness remain hypotheses to be tested in future studies.

## 7. Conclusions and Future Work

This paper introduced and experimentally validated a containerized edge architecture based on visual workflow orchestration for IIoT. By deploying the n8n low-code engine within a lightweight microservices stack on a Raspberry Pi 4, we demonstrated that visual orchestration tools, traditionally associated with business process automation, can operate as reliable middleware in resource-constrained industrial settings.

The proposed Dual-Path routing strategy proved effective in addressing the throughput limitations imposed by public cloud APIs through rate-limiting mechanisms, while preserving full local persistence at the edge. Within the scope of the reported experiments, the paper demonstrates telemetry orchestration, filtering, and selective reporting; it does not demonstrate advanced multi-source data fusion, protocol-rich interoperability, or comparative superiority over alternative middleware stacks.

Across the experimental scenarios, the system maintained a processing latency of approximately 5 ms on average, with observed peaks below 10 ms, placing the solution within the operational bounds of soft real-time industrial monitoring. The edge-side filtering mechanism reduced outbound traffic by 98.0%, enabling full-fidelity local persistence while avoiding the severe throttling observed in the cloud-only reference pipeline, whose rejection ratio reached 98.0% (95% Wilson confidence interval: 97.43–98.44%). These results support the claim that containerized visual orchestrators can provide a maintainable and cost-conscious fog-computing layer for high-frequency telemetry workloads on low-cost hardware.

Despite its promising performance, the study also leaves several hypotheses untested. In particular, the present manuscript does not yet provide an empirical head-to-head comparison against Node-RED or other baselines, does not measure the latency overhead of TLS and authentication on the Raspberry Pi 4, and does not validate resilience through orchestrator-crash fault injection. The most important next step is therefore to convert the present feasibility study into a comparative, security-hardened, and resilience-oriented benchmark. Future work will extend the architecture in four main directions:

1. Security Hardening: Integrating Transport Layer Security (TLS) across the internal Docker network and MQTT communication channels, disabling anonymous broker access, and moving cloud credentials to a proper secret-management mechanism in order to quantify the impact of encryption and authentication on latency and CPU utilization on the Raspberry Pi 4.
2. Comparative Baselines: Benchmarking the same workloads against Node-RED, custom Python/Go services, and MQTT–Telegraf–InfluxDB pipelines to determine the relative cost of low-code orchestration in terms of latency, throughput, CPU, and RAM consumption.
3. Edge Hardware Generalization: Assessing performance on additional platforms, including more constrained ARM devices and x86 industrial PCs, to establish how far the present findings generalize beyond the Raspberry Pi 4.
4. Protocol Diversity and High Availability: Extending the workload to Modbus and OPC UA sources while exploring multi-node orchestration (e.g., clustered brokers, replicated workflow engines), explicit orchestrator-crash fault injection, and recovery-time measurements to provide fault tolerance and evaluate the behavior of visual low-code tools under distributed edge–fog topologies.

By addressing these aspects, future research will contribute to a more comprehensive understanding of the role of low-code workflow engines in next-generation industrial edge infrastructures. In its current form, the paper should therefore be read as evidence that n8n-based edge orchestration is feasible and practically useful for telemetry routing under cloud throttling, not as definitive proof of generalized superiority or full production readiness.

## Figures and Tables

**Table 1:** Qualitative positioning of representative low-code platforms for IIoT edge telemetry.

Platform	Primary Execution Model	Strengths for IIoT Integration	Main Limitations for the Studied Edge Scenario
Node-RED	Flow-based, event-driven runtime on Node.js	Large ecosystem of industrial nodes, strong Raspberry Pi adoption, proven edge suitability in prior benchmarking	Complex flows can become visually dense; the literature emphasizes edge feasibility, but not specifically a hot/cold design for rate-limited cloud reporting
Power Automate	Managed cloud flows and desktop automation	Rich SaaS connectivity, enterprise governance, low barrier for office-process automation	Cloud-first execution model, limited suitability for disconnected edge gateways, and weaker fit for sustained local telemetry persistence
n8n + containerized dual-path stack	Self-hosted workflow orchestration with isolated microservices	Structured workflow logic, explicit credential handling, portable deployment, direct coexistence of local persistence and selective cloud forwarding	Fewer published IIoT edge benchmarks than Node-RED; performance and protocol diversity still require broader validation

**Table 2:** Security roadmap for production deployment.

Control	Planned Implementation	Expected Impact
TLS	Enable TLS for MQTT broker listeners and HTTPS for external software-as-a-service endpoints; manage certificates through mounted secrets or a dedicated secret store.	Higher CPU usage and slightly higher end-to-end latency due to handshake and encryption overhead.
Authentication and authorization	Disable anonymous MQTT, enable username/password or certificate- based identities, and define topic-level access control lists.	Improved tenant and device isolation; minor broker-side authorization overhead.
Secret management	Move API tokens and webhook credentials from plain environment variables to Docker secrets or an external vault-backed mechanism.	Lower credential exposure risk and better rotation discipline.
High availability	Persist MQTT sessions, enable broker persistence, and evaluate replicated workflow engines or standby orchestrators for fail-over.	Improved resilience at the cost of additional orchestration complexity and storage synchronization.

**Table 3:** Performance metrics used in the evaluation.

Metric	Formula/Unit	Interpretation
Processing latency	$L_{proc}=t_{egress}-t_{ingress}$, ms	Measures the Hot Path orchestration overhead between MQTT ingestion and persistence in InfluxDB.
Accepted cloud throughput	$T_{cloud}=N_{accepted}/\Delta t$, req/min	Captures how many requests the external API actually accepts under load.
Cloud rejection ratio	$Q=N_{429}/N_{req}$	Quantifies rate-limit saturation for the cloud-only reference pipeline.
Edge filtering ratio	$R=1-N_{cloud}/N_{in}$	Quantifies how effectively the Cold Path reduces outbound traffic.
Local retention ratio	$P=N_{local}/N_{in}$	Measures whether all telemetry is durably preserved at the edge.
CPU and RAM usage	%, MB	Characterizes the hardware cost of maintaining the containerized stack on the gateway.

**Table 4:** Summary of experimental scenarios.

Scenario	Sensors	Ingress	Architecture	Purpose
A	10 at 1 Hz	600 msg/min	Dual-Path	Baseline latency and functional validation.
B	50 at 1 Hz	3000 req/min	Cloud-only	Rate-limit bottleneck characterization.
C	100 at 0.5 Hz	3000 msg/min	Dual-Path	Stress test with identical ingress and selective cloud forwarding.

**Table 5:** Quantitative summary of the experimental results.

Scenario	Primary Metric	Observed Value	Interpretation
A	Mean Hot Path latency	≈5 ms	Compatible with soft real-time monitoring workloads.
A	Maximum observed Hot Path latency	<10 ms	No visible burst-driven instability in the observed run.
B	Cloud requests generated	3000 req/min	Workload exceeds the software-as-a-service budget by two orders of magnitude.
B	Accepted cloud throughput	≈60 req/min	Endpoint saturates at the practical rate-limit ceiling.
B	Cloud rejection ratio	98.0% (95% CI: 97.43–98.44%)	Direct cloud upload is not sustainable under moderate IIoT fan-in.
C	Local retention ratio	100% observed	All telemetry remained available at the edge through the Hot Path.
C	Edge filtering ratio	98.0%	Cold Path traffic remained within the external API budget.
C	Outbound cloud throughput	≈60 events/min	Selective reporting matches the accepted budget without triggering throttling.

## Data Availability

The data supporting the findings of this study are available from the author upon reasonable request. No publicly archived dataset was used in this work.
